# Supplementary material for: Estimated incidence and clinical presentation of Noma in Northern Nigeria (1999–2024)
Source: PLoS Negl Trop Dis. 2025 May 29;19(5):e0012818. doi: 10.1371/journal.pntd.0012818 (PMC12143531; doi:10.1371/journal.pntd.0012818)
Supplement: Attachment — Submitted filename: Response to reviewers.docx [file pntd.0012818.s002.docx]

**Response to Reviewer’s comments**

**Reviewer 1**

- Braimah and co-authors reviewed a long case series of noma patients from the world’s premier noma hospital and tried to estimate incidence figures and morbidity variables stratified by state across northern Nigeria. This is an important study as few reliable estimates on noma exist, and even less depicting regional clustering and variations in morbidity indicators. The findings put forward by the team confirm some long-held assumptions, e.g. the link with poverty and age, but also add new elements such as the considerable number of older noma patients, some of them presumably presenting for treatment long after surviving noma.

A number of comments are offered for consideration in a further refined and revised version:

- general comment: the analysis is based on hospital data and as such, is inherently biased as only patients with access to the hospital could be included. The incidence estimates try to correct for under-reporting but this assumes homogenous access issues which clearly is not the case – distance certainly plays an important role. Also, no correction for morbidity indicators are used while presumably, the clinical picture of patients who died before reaching the hospital might have been different compared to the cohort that was included in the survey. The implications should be discussed in detail.

Response: Thank you so much for this comment. In the revised manuscript, we tried to account for distance to NCH in the incidence estimation. Also, we have modified the limitations section to include that the demographics and clinical presentation of patients included in our analysis may be significantly different from those that died which out cohort does not capture.

- abstract: at the beginning, noma is clearly described as a disease predominantly affecting young children while the results point to a considerable number of also older noma patients. This may be confusing, and it should be made clear that acute noma primarily occurs in young children while arrested noma can affect individuals of any age.

Response: Thank you for the comments. We have revised the abstract according to your comments.

- introduction: the geographic direction of east and west has been reversed in the description of noma distribution

Response: Thank you for this comment. The statement has been removed from the introduction following our revision.

- methods: should be written in past tense throughout the paragraph (e.g. involved instead of involves)

Response: Thank you for this comment, we have revised the manuscript accordingly.

- methods: why was a clearly outdated census used as population reference?

Response: Thank you for the comment. The last census conducted in Nigeria was in 2006 and those are the latest official population figures available to us for this study. Also, it falls in the middle of the study period, that was why we used it for incidence estimation.

- results: when mentioning incidence, it should always be made clear whether the incidence refers to a period of several years or it is per year.

Response: Thank you for this comment. We have clarified in the manuscript by using terms such as 26-year incidence and annual incidence.

- discussion: reasons for the sharp increase in noma cases over the past 5 years should be discussed as it follows a long period with relatively stable and much lower numbers (increase awareness and community sensitization and case presentation?) Entry of MSF and new stakeholders?

Response: Thank you for the comments. We have included an explanation for the sharp increase in the discussion of the revised manuscript as advised.

- no data on risk factors are presented beyond gender and age. Were they not available or analyzed? If available, at least selected indicators might be added as they may be important to understand temporal and regional dynamics.

Response: Thank you for your comments. Many of the patients are surgical patients and risk factor information were not collected routinely. We were able to gather some information on caregivers and family settings, but they do not seem reliable. As such, the authors decided not to present the data in this study.

**Reviewer 2**

The conclusions are supported. I recommend discussion of limitations, which are not included. The public health relevance is addressed

Response: Thank you for your comment. We have now included a more detailed discussion of limitations.

Thank you to the authors for your research and manuscript on noma in northern Nigeria during the last two and a half decades. The title accurately describes the manuscript.

The authors performed a retrospective study of noma patients who presented to the Noma Children’s Hospital. Their study features the clinical presentation and estimates the incidence of noma in northern Nigeria. With the high and rising incidence of acute noma, the authors urge screening and treatment of acute noma throughout this region.

The abstract is well written. The first sentence could be revised. Noma is not a new tropical disease. As reported in the discussion section, noma is the most recent addition to the WHO list of Neglected Tropical Diseases (NTDs). Noma was included in a textbook of neglected diseases 400 years ago (Affectibus omissis’(Arnoldus Bootius, 1649, Neglected Diseases)

Response: Thanks a lot for your comments. We have revised the sentence accordingly.

This research is limited to patients who presented to the Noma Children’s Hospital. Children with noma may not be recognized or able to travel to the hospital. Estimates are that only 10-30% of patients with acute noma receive treatment. The authors report that more patients presented in Stage 3 than in earlier stages, which may indicate late recognition or difficulties reaching the hospital. Due to the rapid progression of noma and high mortality, the true incidence may be much higher. Please discuss this limitation.

Response: Thank you for this comment. We have followed your recommendation in the manuscript. Also, the high mortality rate of noma was considered during incidence estimation in this study.

The last sentence in the introduction includes “genetic predisposition” as one of the noma risk factors. However, the references do not support this point, suggesting only that periodontal diseases may have genetic predispositions. Extreme poverty, chronic malnutrition, poor hygiene, underlying diseases or recent acute illness, and lack of dental care may compromise the patient’s immune system, resulting in microbial dysbiosis and susceptibility to noma.

Response: Thank you for the clarification and comments. We have revised the manuscript in line with your recommendation.

Do the authors think the high incidence of noma cases in Sokoto is influenced by the presence of the Noma Children’s Hospital?

Response: Thank you for the question. We believe this may be one of the factors and explains the result but only to an extent. Of note, we also observed some clustering in the high incidence of noma in north west Nigeria in the Sokoto-Kebbi-Zamfara axis. These areas are among states with high poverty rates in Nigeria and that may also explain the reason why NCH was even established in Sokoto in the first place.

Regarding the last sentence of Data Collection, please correct the statement about acute and “arrested noma.” According to WHO stages, Stages 1 & 2 are reversible with treatment. Stages 3-5 are irreversible, and efforts are required to save the patient’s life. Even without treatment, some noma patients survive, but it is not known why their disease is arrested. Patients with “arrested noma” still progress through all the stages, including scarring and sequelae.

Response: Thank you for this observation. We have included the reversible and irreversible stages as advised. In the study, we only coined acute noma/active disease to showcase stages 1 to 3 and “arrested noma” or noma sequalae to depict stages 4 and 5.

Thank you to the authors for your research on noma patients in Nigeria. This research should lead to a better understanding of this neglected disease and encourage effective interventions to eradicate this preventable childhood disease.

**Reviewer 3**

The objective of the study is mostly clear. There is no hypothesis, but as the objective is exploratory, I do not think it is necessary.

The study design has been adapted from previous studies (i.e. the WHO Delphi consultation). In my personal opinion, it would be interesting to try to calculate incidence by considering the population at risk, if the population data is available. However, if not the method can be used to a certain degree. One big limitation is that the formula is based on the proportion of surviving cases that have been referred. This number will differ greatly across states. For example, the proportion of surviving cases reaching the hospital in Sokoto who are originally from Sokoto will be much bigger compared to the proportion of cases reaching Sokoto from Adamawa state, which is on the other side of the country. Ideally, these different proportions should be estimated and adapted for the different states that are represented. If not, it should at least be critically reflected in the discussion as a major limitation. States cannot be compared as it is, otherwise, we don’t know if the lower incidence the further a state is away from Sokoto is caused by the smaller population reaching Sokoto or a depiction of reality.

Response: We appreciate your insight on the incidence estimation which led us to revise the methodology to factor in distance to NCH in selecting the proportion of cases that reach the hospital. In the revised manuscript. We first calculate the incidence at the state-level for all analysis then add the number of new cases in all states for overall incidence estimation. Also, for incidence estimation by sex and age group, we used the population at risk as denominator instead of the general population.

Another point where we need to be careful is that 2 states have been excluded because no noma cases have been reported. If we do this, we cannot speak of an overall incidence across Northern Nigeria. By not including states without noma, we probably overestimate the incidence. If these states are not considered, the results need to be presented precisely and this issue needs to be discussed in the discussion.

Response: Thank you for this comment. We have followed your recommendation and included the population of Nasarawa and plateau states for incidence estimation.

In general, the results are very comprehensive and interesting.

When reporting on age groups, I recommend to always differentiate between active and arrested noma (also in the text), otherwise we risk to confuse people about the age of noma onset. In general, in the demographic table, it would be nice to have a row of age of noma onset, if the data is available.

Response: Thank you for the comment. We have differentiated between active disease and arrested noma when describing the age groups throughout the manuscript. Also, the age on onset data is not available for those with arrested noma and was not considered in our study.

In the results tables, it is not always clear which comparisons the p-values concern. E.g. in table 1 we do not know if the p-value stems from a test comparing age groups or active and passive disease. If possible, it would be nice to see 95% Confidence intervals.

Response: Thank you for your comment. The comparison is between patients with active disease and arrested noma based on their demographic characteristics e.g. age, sex etc. We have clarified the meaning of these comparisons as footnotes of the table in the manuscript. Also, confidence intervals have been included for the median ages in Table 1.

Language-wise, I recommend to be careful on how incidence and number of patients are presented. As it is written now, it might sometimes give the impression that the results represent the whole area or population groups, but the whole analysis is based on people who reached Sokoto noma hospital. (Estimated incidence) For example, “the analysis showed that more patients had gangrene than oedema or ANUG” could be clearer like this “the analysis showed that more patients were admitted to the noma hospital with gangrene compared to oedema or ANUG”.

For incidence it is not always clear in the text if it concerns the study period or an average.

Response: Thank you for your comments. We have revised the language in the manuscript based on your comment. We have also clarified the reporting of incidence in the manuscript by adding whether it concerned annual incidence or incidence within the study period (26 years).

The discussion could be less focused on the results but more critical about limitations. E.g. address difference in proportion of people reaching Sokoto depending on their geographic location, retrospective nature of study, is the study representative, do higher incidence rates in recent years really represent higher incidence or could they be caused by a bigger awareness of noma in Nigeria due to the engagement of the MoH and NGOs, consequently more people visit the Sokoto hospital, etc.

Response: Thank you for this comment. We have improved on the discussion and presentation of the study limitations based on your comments.

The conclusions mainly repeat the results but do not reflect on how this study advances our understanding of noma or its public health relevance. Even though it is a very relevant study with extremely valuable data.

Response: Thank you for the comments. We have revised the conclusion to include the public health relevance of the study.

I highly discourage speaking of a "noma belt" or noma as an "African problem". We have most reports and noma data from the African region, but also most studies on noma were conducted there. Recent evidence suggests that noma is a global problem, wherever extreme poverty exists. By always focusing on the noma belt/the African continent, we discourage other regions to start looking for noma cases.

Response: Thank you for your comments. We have removed the term in the manuscript according to your recommendations.

In figures 1&2 please clearly state that these are the noma cases registered in the noma hospital to avoid giving the impression that these are absolute and representative numbers.

Response: Thank you for the comment. The caption has been revised accordingly.

Figures 5&6: you are speaking of noma incidence, hence referring to stages 1-3. In the results tables, the higher age groups did not present in stages 1-3, so I am not sure why they are presented in the incidence figures.

Response: Thank you for your comments. In the study, 931 persons had noma stages 1-3 at presentation and this data was used for incidence estimation. All the persons with higher age group in the incidence results had acute noma at presentation which is one unique findings our study reports. Arrested noma (stages 4 and 5) include 452 cases and data was not used for incidence estimation at all.

Adapting the proportion of patients reaching the hospital based on geographic distance from Sokoto.

Response: Thank you! We have followed this recommendation

2. **Re-integrating the states without any noma cases in your analysis** or clearly discussing the implications of excluding them on your results.

Response: Thank you! We have followed this recommendation

Critically reflect your limitations in the discussion.

Response: Thank you! We have followed this recommendation

Rewrite the conclusion section to indicate how your study contributed to noma research and public health and what this means. E.g. do we need further studies, does this help us to better design interventions (e.g. most patients arrive at stage 3, how can we reach them in earlier stages), do you have recommendations.

Response: Thank you so much! We have followed your comments in refining the conclusion section.
